# A reinvestigation of somatic hypermethylation at the *PTEN *CpG island in cancer cell lines

**DOI:** 10.1186/1480-9222-14-5

**Published:** 2012-04-10

**Authors:** Luke B Hesson, Deborah Packham, Emily Pontzer, Pauline Funchain, Charis Eng, Robyn L Ward

**Affiliations:** 1 Adult Cancer Program, Lowy Cancer Research Centre and Prince of Wales Clinical School, University of New South Wales, Sydney, NSW 2052, Australia; 2 Genomic Medicine Institute, Lerner Research Institute, Cleveland Clinic, Cleveland, OH 44195, USA; 3 Department of Genetics and CASE Comprehensive Cancer Center, Cleveland, OH 44116, USA

**Keywords:** DNA methylation, Epigenetic, *PTEN*, *KILLIN*, *PTENP1*, Pseudogene, Cowden syndrome

## Abstract

**Background:**

*PTEN *is an important tumour suppressor gene that is mutated in Cowden syndrome as well as various sporadic cancers. CpG island hypermethylation is another route to tumour suppressor gene inactivation, however, the literature regarding *PTEN *hypermethylation in cancer is controversial. Furthermore, investigation of the methylation status of the *PTEN *CpG island is challenging due to sequence homology with the *PTEN *pseudogene, *PTENP1. PTEN *shares a CpG island promoter with another gene known as *KLLN*. Here we present a thorough reinvestigation of the methylation status of the *PTEN *CpG island in DNA from colorectal, breast, ovarian, glioma, lung and haematological cancer cell lines.

**Results:**

Using a range of bisulphite-based PCR assays we investigated 6 regions across the *PTEN *CpG island. We found that regions 1-4 were not methylated in cancer cell lines (0/36). By allelic bisulphite sequencing and pyrosequencing methylation was detected in regions 5 and 6 in colorectal, breast and haematological cancer cell lines. However, methylation detected in this region was associated with the *PTENP1 *promoter and not the *PTEN *CpG island.

**Conclusions:**

We show that methylation of the *PTEN *CpG island is a rare event in cancer cell lines and that apparent methylation most likely originates from homologous regions of the *PTENP1 *pseudogene promoter. Future studies should utilize assays that reliably discriminate between *PTEN *and *PTENP1 *to avoid data misinterpretation.

## Background

Phosphatase and tensin homologue (*PTEN*) is a tumour suppressor gene with dual protein and lipid phosphatase activity. The main mechanism by which *PTEN *functions as a tumour suppressor is by negatively regulating the PI3K-AKT-mTOR pathway [1,2]. Inactivating germline mutations of *PTEN *result in a group of rare syndromes collectively known as the *PTEN *hamartoma tumour syndromes (PHTS), which includes Cowden syndrome and Bannayan-Zonana syndrome [3]. Germline mutations of *PTEN *account for approximately 80% of Cowden syndrome cases [4]. This syndrome is characterised by a range of clinical features including an increased risk of breast, thyroid and endometrial cancers [3]. Mutation of the *PTEN *tumour suppressor gene also occurs in various sporadic cancers, including 38% of endometrial, 14% of prostate, 7% of colorectal and 5% of lung carcinomas [5].

Promoter CpG island hypermethylation, which can result in the transcriptional silencing of gene expression, is an alternative mechanism of gene inactivation. The importance of *PTEN *inactivation in PHTS and several types of sporadic cancers makes the gene an attractive candidate for epigenetic inactivation. The *PTEN *CpG island is shared with another gene, known as *KLLN*, which is transcribed from the negative DNA strand in the opposite direction. There are a number of methodological challenges associated with the detection of methylation at the *PTEN *CpG island and in determining the consequences of methylation on the expression of *PTEN*. These challenges arise due to the fact that the *PTENP1 *pseudogene (also known as *PTEN2*), shares 97.8% sequence identity with the *PTEN *mRNA sequence, and 91% identity with a 921 bp region of the *PTEN *CpG island. Consequently, without careful consideration of assay design there is a possibility of amplification of *PTENP1 *rather than the *PTEN *gene. The *PTENP1 *pseudogene is a single exon gene located at 9p13.3 (genomic coordinates chr9:33,663,502-33,667,418, NCBI36/hg18, March 2006 freeze). The existence of the *PTENP1 *pseudogene has been known for some time [6,7] and is thought to have arisen due to retrotransposition to form an intronless copy of the *PTEN *gene. Though some earlier reports suggested *PTENP1 *is not transcribed [7], more recent data suggests that the *PTENP1 *mRNA is ubiquitously expressed in both normal and cancer specimens [8-11]. One study showed that *PTENP1 *mRNA levels may be as high as 70% of *PTEN *mRNA levels [8]. As a consequence of *PTENP1 *expression, accurate analysis of *PTEN *mRNA levels can be problematic and careful consideration of assay design is required. Most previous studies have chosen to analyse *PTEN *expression using immunohistochemistry [12,13], however, *PTEN*-specific RT-PCR assays that utilise sequence variations between the *PTEN *and *PTENP1 *genes have been described [14].

There have been occasional reports of *PTEN *promoter hypermethylation in cancer. Marsit *et al*. reported hypermethylation of a region of the *PTEN *CpG island that is not homologous to *PTENP1 *in 26% (39/151) of non-small cell lung cancers (NSCLC), however promoter methylation was not a strong predictor of reduced or absent *PTEN *protein expression. Interestingly, in the same study, *PTENP1 *promoter hypermethylation was found in 66% (112/169) of NSCLCs, but not in normal peripheral blood DNA [12]. Hypermethylation of two CpG sites within the *PTEN *CpG island was also reported in the peripheral blood of up to 62% of patients with metastatic melanoma [15], however, methylation and expression of the *PTENP1 *gene was not addressed in this study.

Recently, a study using constitutional DNA from 123 Cowden and Cowden-like syndrome patients without *PTEN *mutations reported hypermethylation of a specific region of the *PTEN *CpG island located within the 5' UTR of the *PTEN *gene [16]. Interestingly, this study suggested that hypermethylation of this region correlated with downregulation of *KLLN *expression rather than *PTEN *expression. Inactivation of *KLLN *expression in patients with sporadic renal cell carcinomas by CpG island hypermethylation was later proposed in another study from the same laboratory [17]. Recent evidence suggests the *KLLN *gene is necessary for p53-induced apoptosis and may therefore possess tumour suppressor function [18].

Several studies have described how differences in the sequences of *PTEN *and *PTENP1 *can be misinterpreted as *PTEN *mutations [19]. Furthermore, one previous study demonstrated that methylation of the *PTENP1 *gene can also be misinterpreted as methylation of the *PTEN *CpG island [20]. In the current study we present a thorough reinvestigation of the *PTEN *CpG island to assess whether hypermethylation occurs in cancer and to specifically address some of the methodological challenges associated with the analysis of methylation at this CpG island.

## Results

### Methylation analysis of the *PTEN *CpG island

**Figure 1:**
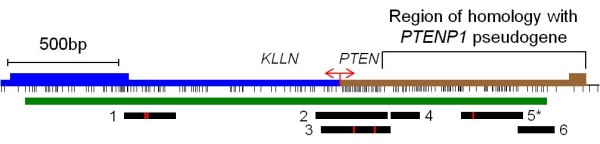
**The *PTEN *CpG island and locations of the regions assayed for methylation in this study**. The *PTEN *CpG island (green bar) encompasses a bidirectional promoter from which the *KLLN *(blue bar) and *PTEN *(brown bar) genes are transcribed from the same transcription start site. The *PTEN *gene is transcribed from the positive strand whereas the *KLLN *gene is transcribed from the negative strand in the opposite direction. The black bars indicate the regions analysed for methylation using bisulphite based PCR assays (see Table 1 for assays, exact position of amplicons and primer sequences). The red vertical lines within the black bars indicate restriction endonuclease sites used to assay for methylation by COBRA. Indicated by the thin black vertical lines are the locations of CG dinucleotides. Also indicated is a region of the *PTEN *CpG island encompassing 921 bp which shows 91% sequence identity with the 5' region of the *PTENP1 *pseudogene located at 9p13.3. * For region 5, primers were taken from Bennett *et al*. [16]. These primers bind sequence that are identical in both the *PTEN *and *PTENP1 *genes producing 283 bp and 291 bp amplicons respectively and the HpyCH4IV restriction site used assay for methylation using COBRA is conserved.

**Figure 2:**
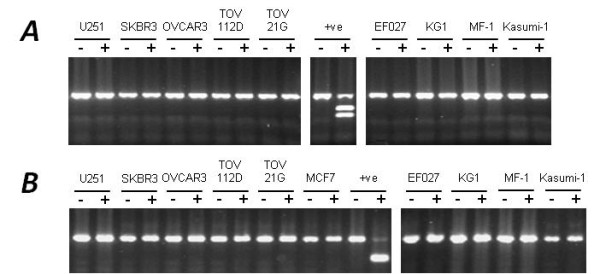
**Methylation analysis of the *PTEN *CpG island using COBRA**. Two regions of the *PTEN *CpG island were investigated for methylation using COBRA and were designated regions 1 and 3 as indicated in Figure 1. ***A***, Region 1 was assayed for methylation using the restriction endonuclease AatII. Identical results were also obtained using the restriction endonuclease ClaI. ***B***, Region 3 was assayed for methylation using the restriction endonuclease TaqI. Identical results were also obtained using the restriction endonuclease MluI. No methylation was found in any sample tested. For each region we used M.SssI *in vitro *methylated normal blood DNA as a positive (+ve) control. -, undigested PCR product; +, digested PCR product.

**Figure 3:**
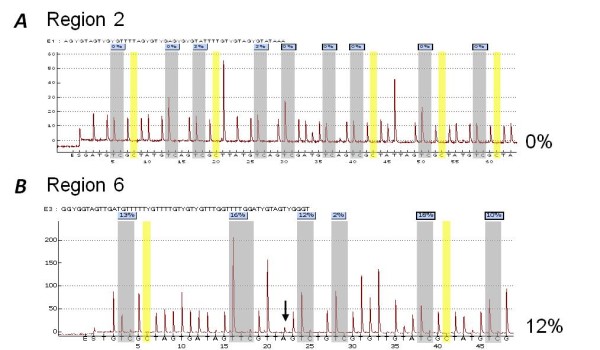
**Bisulphite pyrosequencing analysis of the *PTEN *CpG island**. Shown are representative pyrograms of region 2 (panel A) and region 6 (panel B) of the *PTEN *CpG island from the colorectal cancer cell line RKO. ***A***, Region 2 was completely unmethylated in all samples tested. ***B***, Region 6 showed a mean of 12% methylation across the 6 CpG sites tested in the colorectal cancer cell line RKO. Indicated by the black arrow is the presence of an A nucleotide variant specific to the *PTENP1 *pseudogene suggesting non-specific amplification of *PTENP1*. Blue boxes indicate the percent methylation at each CpG site. Grey shading indicates the CpG sites tested. Yellow shading indicates bisulphite conversion control dispensations, which indicated complete conversion in all samples tested.

**Figure 4:**
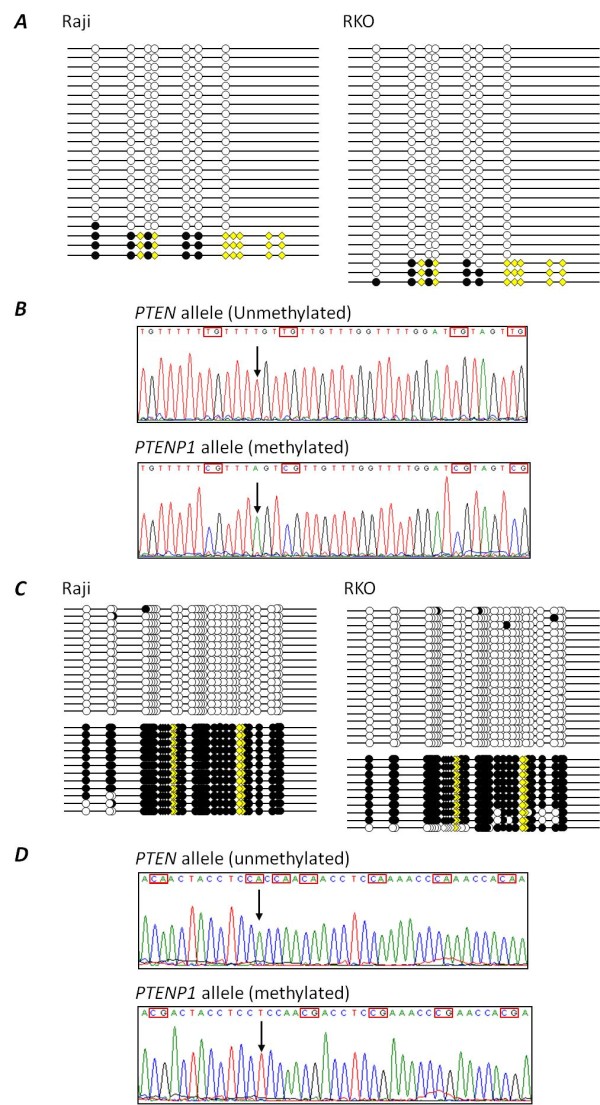
**DNA methylation is specifically associated with *PTENP1 *and not the *PTEN *CpG island**. ***A***, Representative sequencing data of region 6 from the haematological and colorectal cancer cell lines Raji and RKO. Only *PTENP1 *derived alleles showed DNA methylation. Each line represents a single allele. Circles indicate the positions of CpG dinucleotides; black circles indicate methylated CpG dinucleotides; white circles indicate unmethylated CpG dinucleotides; yellow diamonds indicate the positions of nucleotide variations within *PTENP1 *alleles used to discriminate between *PTEN *and *PTENP1 *alleles. ***B***, Representative electropherograms from region 6 showing an unmethylated *PTEN *allele and a methylated *PTENP1 *allele from the colorectal cancer cell line RKO. Indicated by the black arrow is the position of a variant nucleotide which was used to discriminate between *PTEN *and *PTENP1 *alleles (the same nucleotide indicated by the black arrow in Figure 3B). CpG dinucleotides are boxed in red. ***C***, Allelic bisulphite sequencing of region 5 in the cancer cell lines Raji and RKO. Again, only *PTENP1 *derived alleles showed methylation. Sequence variations used to discriminate between *PTEN *and *PTENP1 *alleles are indicated by yellow diamonds. Black diamonds indicate the positions of additional CpG dinucleotides specific to *PTENP1 *alleles that were also methylated. ***D***, Representative electropherograms from region 5 showing an unmethylated *PTEN *allele and a methylated *PTENP1 *allele from the cell line Raji. Indicated by the black arrow is the position of a nucleotide variation used to discriminate the PTEN and PTENP1 alleles.

We designed bisulphite-based PCR assays to determine the methylation status of six regions (designated regions 1-6) within the *PTEN *CpG island. The regions examined for methylation, the position of these regions relative to the *PTEN *and *KLLN *genes, and the type of assay used are described in Figure 1 and Table 1. These regions encompassed the majority of the *PTEN *CpG island and included the *KLLN *and *PTEN *transcription start sites (TSS). Regions 1-4 are not homologous to the *PTENP1 *pseudogene whereas regions 5 and 6 show 97.6% and 95.8% homology with the 5' region of the *PTENP1 *pseudogene. The methylation status of these regions was investigated using a range of methods, each with different sensitivities for methylation detection. These included combined bisulphite restriction analysis (COBRA), allelic bisulphite sequencing, bisulphite pyrosequencing and quantitative real-time methylation-specific PCR (qMSP). Using COBRA we found that the *KLLN *gene body (region 1), as well as the *KLLN *and *PTEN *TSS (region 3) were completely unmethylated in all 36 cancer cell lines analysed (Figure 2). We confirmed these results using allelic bisulphite sequencing (data not shown). Using bisulphite pyrosequencing we then examined nine CpG sites within the region around the TSS of these genes (region 2). This assay also showed that all cancer cell lines were unmethylated (Figure 3A), thus confirming our COBRA and bisulphite sequencing data across the same region. Next we examined region 4 downstream of the *PTEN *TSS using qMSP, which has a methylation detection sensitivity level several orders of magnitude higher than COBRA and bisulphite pyrosequencing. Despite this increased sensitivity we again observed that all 36 cancer cell lines were unmethylated (data not shown). We then examined the regions of the PTEN CpG island most similar in sequence to the promoter region of the PTEN pseudogene (regions 5 and 6). Region 5 was examined using allelic bisulphite sequencing whereas region 6 was examined using a bisulphite pyrosequencing assay encompassing six CpG sites. Interestingly, using these assays we found methylation in five out of eight cancer cell lines (Figure 3B). These cell lines were RKO and SW48 (colorectal), Raji and U937 (haematological) and MDA-MB-231 (breast). However, given the similarity of these regions to the promoter region of the *PTENP1 *pseudogene we next determined whether the methylation observed was attributable to non-specific amplification of the homologous *PTENP1 *region. Indeed, closer inspection of the pyrograms revealed the presence of an adenine nucleotide peak at the exact position of a thymine to adenine transversion specific to the *PTENP1 *pseudogene (shown by the black arrow in Figure 3B). The presence of this adenine peak suggested that *PTENP1 *alleles were also amplified using these assays. To confirm whether this was the case, and to determine whether *PTENP1 *alleles could account for the observed methylation, we performed allelic bisulphite sequencing at both regions. We utilised several single nucleotide sequence differences between *PTEN *and *PTENP1 *within these regions to discriminate *PTEN *alleles from *PTENP1 *alleles. We found that non-specific amplification of the *PTENP1 *pseudogene accounted for up to 44% and 22% of alleles amplified using the assays at regions 5 and 6 respectively. Furthermore, methylation was specifically associated with *PTENP1 *alleles whereas all *PTEN *alleles were unmethylated (Figure 4).

**Table 1:** Primer sequences, regions examined for methylation, and the methods used to detect methylation

Region	Method	Position	Size (bp)	Forward primer (5'-3')	Reverse primer (5'-3')	Pyrosequencing primer (5'-3')
1	COBRA Bisulphite sequencing	-966 to -733	233	AGTTTGGTTTYGGGYGATTTAT TTTGT	CCCAAAAAACACCTATCTAAATA AACT	

2	Bisulphite pyrosequencing	-110 to +217	327	TTATGGTTGTAGTTTTYGAGAG GAGAGAAT	CTACAAAAACCRCAACAAATAC AACTACAAACTAA (round 1) CCCAAACAACTACACTAAACAT ACTCAA (nested)	GAGAGGAGAGAATTG

3	COBRA Bisulphite sequencing	-80 to +234	314	TTTAGTAGAGTTTGTGGTTTGG GGATTT	CAAACTTCCATCATAACTACAA CTTCC	

4	qMSP	+237 to +368	132	AGTTGAGTCGTTGTGAGGCGA GGTCG	CAAACCGACCGACTCCCCGAAAACG	

5	Bisulphite sequencing	+554 to +836	283	GTTGTAGTTTTAGGGAGGGGGT	CRCCRCTACCAAACCTCTAACTACTA	

6	Bisulphite sequencing Bisulphite pyrosequencing	+801 to +971	171	GGTTTTTTTTGTAGGATGGAAA TGGT	CRCCRCTACCAAACCTCTAACTACTA	AAATGGTTTTGGATTT

Six regions of the *PTEN *CpG island were investigated for methylation in this study using various methods. Listed are the regions assayed, the method used for the detection of DNA methylation, the position of the region analysed relative to the transcription start site of the *PTEN *gene (NM_000314) located at Chr10:89613175 (NCBI36/hg18, March 2006 freeze), the size of each amplicon, and primer sequences (including pyrosequencing primer sequences)

## Discussion

In this study, we present a thorough reinvestigation of the methylation status of the *PTEN *CpG island in an extensive cohort of cancer cell lines. Using a range of methods including COBRA, bisulphite pyrosequencing, allelic bisulphite sequencing and qMSP we show that hypermethylation of this CpG island in cancer cell lines is rare. These techniques allow the detection of methylation within individual alleles, as well as the quantitative measurement of methylation at individual CpG dinucleotides with extremely high sensitivity. Despite this meticulous analysis, all cell lines analysed were unmethylated. Our assays spanned the majority of the *PTEN *CpG island including the *PTEN *and *KLLN *TSS, the *PTEN *and *KLLN *gene bodies, as well as regions previously reported as hypermethylated [16,17]. We utilised allelic bisulphite sequencing and sequence variations between the *PTEN *and *PTENP1 *gene to unequivocally demonstrate that apparent methylation of the *PTEN *CpG island is actually attributable to the non-specific amplification of the highly homologous *PTENP1 *gene.

The challenges posed by sequence homology with pseudogenes are by no means particular to the *PTEN *gene. Another example is the DNA mismatch repair gene postmeiotic segregation increased 2 (*PMS2*) gene, which shares at least 95.2% sequence identity with six other genes (*PMS2CL, PMS2L2, PMS2P4, PMS2P5, PMS2P1, PMS2P11*) as well as a further 10 sites, all on chromosome 7. Importantly, the *PMS2 *CpG island shares over 87.5% sequence identity encompassing at least 849 bp of the 5' regions of the *PMS2L5, PMS2P4, PMS2L2 *and *PMS2P1 *genes. This makes the methylation analysis of the *PMS2 *CpG island particularly challenging.

There are a number of methodological strategies that can be employed to prevent the misinterpretation of data when investigating the methylation status of a gene with a pseudogene. When designing assays for a given gene, the existence of a pseudogene or other regions of high sequence homology should first be determined using *in silico *sequence analysis of the mRNA and CpG island sequences. Following sequence comparisons assays should utilise critical sequence variations that allow the specific amplification of the gene of interest, or regions of high homology can be avoided completely. Newly designed assays must always be validated using methods that can discriminate between individual alleles. This is most easily achieved using allelic bisulphite sequencing. It is also crucial to be aware of amplicon sizes given that multiple amplicons or unexpected amplicon sizes can indicate non-specific amplification of related sequences such as pseudogenes. Finally, we recommend that the methylation status of regions with particularly high homology to pseudogenes can only be determined using allelic bisulphite sequencing to allow the identification of pseudogene-specific alleles.

## Conclusions

We show that somatic hypermethylation of the *PTEN *CpG island is rare in cancer cell lines. Our study demonstrates the technical challenges associated with analysing the methylation status of the *PTEN *CpG island and suggests methodological strategies for studying the methylation status of genes with pseudogenes, such as *PTEN*. It is imperative that the methodological challenges outlined in this study are taken into account when interpreting the data from previous and future studies of the *PTEN *CpG island.

## Methods

### Sample details and DNA extraction

DNA from 36 cancer cell lines was extracted using the standard phenol-chloroform method. These included 20 colorectal (RKO, SW48, HT-29, Lim1215, SW480, SNU-C2B, Lim2412, SW620, Lim2405, DLD1, HCT116, HCT-15, LoVo, LS147T, Colo 320 DM, LS411, Caco-2, SW948, HCT8, COLO 205), 3 breast (MCF-7, SK-BR-3, MDA-MB-231), 6 haematological (Raji, U937, K562, KG1, MF-1, Kasumi-1), 4 ovarian (OVCAR-3, TOV-112D, TOV-21 G, EF027) one glioma (U251), one embryonal carcinoma (NCCIT) and one lung (A549) cancer cell line

### Sodium bisulfite modification

Genomic DNA was treated with sodium bisulphite using the EZ DNA Methylation-Gold kit (Zymo Research, Orange, CA, USA), according the manufacturers' instructions. A measure of 1-1.5 μg DNA was incubated for 10 min at 98°C, followed by 18 hr at 53°C. Bisulphite converted DNA was eluted in 20-25 μL M-elution buffer and stored at -20°C until required.

### Primer sequences

All primer sequences used throughout this study are listed in Table 1.

### Combined bisulphite restriction analysis

The methylation of two regions of the *PTEN *CpG island (regions 1 and 3, Table 1 and Figure 1) were analysed by COBRA, consisting of sodium bisulphite modification followed by PCR and restriction digestion [21]. For each assay, the PCR mixture contained 1x PCR buffer, 0.25 mM each dNTP, 1.5 mM MgCl_2_, 0.4 μM forward and reverse primers (Geneworks), and 1.25 U Platinum *Taq *polymerase (Invitrogen), and 80-225 ngbisulphite modified DNA. The PCR mixture for region 1 was subject to thermocycling at 95°C for 5 min, 40 cycles of 95°C for 30 sec/51°C for 30 sec/72°C for 30 sec, followed by a final extension of 72°C for 10 min. The PCR mixture for region 3 was subjected to thermocycling at 95°C for 5 min, 10 cycles of touchdown at 95°C for 30 sec/65°C for 30 sec/72°C for 30 sec, 30 cycles at 95°C for 30 sec/55°C for 30 sec/72°C for 30 sec, followed by a final extension of 72°C for 10 min. The PCR mixture for region 3 was subjected to thermocycling at 95°C for 5 min, 45 cycles of 95°C for 30 sec/62°C for 30 sec/72°C for 30 sec, followed by a final extension of 72°C for 10 min. Prior to restriction digestion 3 μL of each amplicon was resolved on a 1.5% agarose gel for quality control. Restriction digest reactions were carried out in a total volume of 15 μL containing the appropriate 1x restriction enzyme buffer, 1x BSA if required, 10 U restriction enzyme (New England Biolabs) and 9.5 μL amplicon. To assay for methylation the restriction enzymes AatII and ClaI (region 1) or TaqI and MluI (region 3) were used, each in separate reactions. For each restriction digest, an equal amount of amplicon was subject to the same conditions without the addition of restriction enzyme. Digest reactions with and without restriction enzyme were resolved in adjacent lanes on a 2% high resolution agarose gel to prevent background bands from the PCR reaction from being misinterpreted as digestion, and therefore methylation. Each assay included a fully methylated positive control (genomic DNA from a normal individual methylated with CpG M.SssI methylase), as well as normal blood DNA.

### Quantitative real-time methylation specific PCR

Quantitative real-time methylation-specific PCR was used to assay for methylation at region 4 (Table 1 and Figure 1). This assay consists of sodium bisulphite modification followed by PCR with primers specific to methylated template. The PCR mixture contained 1x iQ™ SYBR Green^® ^Supermix buffer (Bio-Rad), 0.3 μM forward and reverse primers (Geneworks) and 80-120 ng bisulphite modified DNA. This PCR mixture was subjected to thermocycling at 95°C for 2 min, 42 cycles at 95°C for 30 sec/70°C for sec/72°C for 30 sec, followed by melt curve analysis from 70°C to 94°C. In addition, a bisulphite specific control reaction using *MyoD *measured the loading of bisulphite converted DNA. The *MyoD *reaction is not methylation specific but is specific for bisulphite converted DNA. The *MyoD *specific primers used were 5'- CCAACTCCAAATCCCCTCTCTAT-3' and 5'-TGATTAATTTAGATTGGGTTTAGAGAAGGA-3'. *MyoD *MSP was subject to thermocycling at 95°C for 2 min, 42 cycles at 95°C for 30 sec/58.5°C for 30 sec/72°C for 30 sec/77.5°C for 30 sec, followed by melt curve analysis from 70°C to 88.5°C. Absolute values for experimental samples were calculated from the PCR cycle number at which the fluorescence crossed the threshold with the use of the standard curve. Values were normalised against *MyoD *and the percentage of methylated alleles was calculated with reference to 100% *in vitro *methylated human DNA (Zymo Research, Orange, CA, USA).

### Pyrosequencing

We used pyrosequencing to assay for methylation at two regions of the *PTEN *CpG island (regions 2 and 5, Table 1 and Figure 1). Pyrosequencing consisted of sodium bisulphite modification followed by PCR and pyrosequencing with an additional internal primer. In all cases the reverse primer was labelled with biotin. For region 2 we used semi-nested PCR. Both rounds of PCR contained 1x PCR buffer, 0.25 mM each dNTP, 1.5 mM MgCl_2_, 0.4 μM forward and reverse primers and 1.25 U Platinum *Taq *polymerase, and 80-120 ng bisulphite modified DNA. This PCR mixture was subjected to thermocycling at 95°C for 5 min, 30 cycles at 95°C for 30 sec/61°C for 30 sec/72°C for 30 sec, followed by a final extension of 72°C for 10 min. For the second round, 1 μL of the first round PCR was added to a second PCR reaction containing the same ingredients but with a nested reverse primer and subjected to the same thermocycle as above. For region 5, the PCR mixture contained 1x PCR buffer, 0.25 mM each dNTP, 1.5 mM MgCl_2_, 0.4 μM forward and reverse primers and 1.25 U Platinum *Taq *polymerase, and 80-120 ng bisulphite modified DNA. This PCR mixture was subjected to thermocycling at 95°C for 5 min, 45 cycles of 95°C for 30 sec/61°C for 30 sec/72°C for 30 sec, followed by a final extension of 72°C for 10 min. Prior to pyrosequencing 3-5 μl of amplicon were checked on a 1.5% agarose gel. For pyrosequencing, biotinylated amplicon were first immobilized using streptavidin-coated sepharose beads (Amersham Biosciences) and binding buffer according to the manufacturer's instructions. The strands were separated using a Vacuum Prep Station (Biotage), and diluted in 40 μl annealing buffer containing 0.4 μM sequencing primer. The sequencing primers (Table 1) were annealed to the template at 90°C for 2 min. For region 2, the nucleotide dispensation order used was GATGTCGCTATGTCAGTCGCTTATGTCAGTCGATGTCAGTCGCTATTAGTCGCTATGTCGCTA. For region 5, the nucleotide dispensation order used was TGTCGCTAGTGATAGTTCGTTAGTCTGTCGTGTTGTATCGCTATGTCG. Pyrosequencing was performed using the PyroGoldReagents (Biotage) and Pyromark ID system (Biotage). The software PyroMark CpG Software (version 1.0) was used for evaluation of peaks for methylation by SQA analysis.

### Allelic bisulphite sequencing

Individual alleles were cloned and bisulphite sequenced for regions 1, 3, 5 and 6. Amplicons were cloned into the pCR^®^2.1-TOPO^® ^vector (Invitrogen) and transformed into TOP10 One Shot Cells or DH5α One Shot Cells (Invitrogen) according to the manufacturers' instructions. Following transformation and selection of bacteria on agar plates we performed colony PCR using M13 primers. The PCR mixture contained 1x PCR buffer, 0.25 mM each dNTP, 2.5 mM MgCl_2_, 0.4 μM M13 forward and M13 reverse primers and 1.25 U Platinum *Taq *polymerase, and inoculated with part of a discrete bacterial colony. This PCR mixture was subject to thermocycling at 95°C for 5 min, 35 cycles of 95°C for 30 sec/52°C for 30 sec/72°C for 30 sec, followed by a final extension of 72°C for 10 min. PCR products were purified using MultiScreen PCR plates (Millipore) and then subject to sequencing using the ABI BigDye Cycle Sequencing kit (Perkin Elmer) using the M13 reverse primer. Following ethanol precipitation of the sequenced products, samples were analysed using an ABI 3730 DNA sequencer (Perkin Elmer).

## Abbreviations

COBRA: Combined bisulphite restriction analysis; NSCLC: Non small cell lung cancer; *PTEN*: Phosphatase and tensin homologue; *PTENP1*: *PTEN *pseudogene 1; PHTS: *PTEN *hamartoma tumour syndrome; qMSP: Quantitative real-time methylation-specific polymerase chain reaction; RT-PCR: Reverse transcriptase polymerase chain reaction; TSS: Transcription start site; UTR: Untranslated region.

## Competing interests

The authors declare that they have no competing interests.

## Authors' contributions

DP and LBH were involved in assay design, as well as acquisition and analysis of data. LBH and RLW were involved in the study concept, and interpretation of the data. EP, PF, and CE provided expertise with methodology. RLW and LBH obtained funding. All authors were involved in drafting and critical revision of the manuscript and gave final approval of the version published. All authors read and approve the final manuscript.
